# Ion Transport in Eryptosis, the Suicidal Death of Erythrocytes

**DOI:** 10.3389/fcell.2020.00597

**Published:** 2020-07-08

**Authors:** Michael Föller, Florian Lang

**Affiliations:** ^1^Department of Physiology, University of Hohenheim, Stuttgart, Germany; ^2^Department of Physiology Institute of Physiology, University of Tübingen, Tübingen, Germany

**Keywords:** phosphatidylserine, Cacpsdummy2+, shrinkage, gardos channel, apoptosis

## Abstract

Erythrocytes are among the most abundant cells in mammals and are perfectly adapted to their main functions, i.e., the transport of O_2_ to peripheral tissues and the contribution to CO_2_ transport to the lungs. In contrast to other cells, they are fully devoid of organelles. Similar to apoptosis of nucleated cells erythrocytes may enter suicidal death, eryptosis, which is characterized by the presentation of membrane phosphatidylserine on the cell surface and cell shrinkage, hallmarks that are also typical of apoptosis. Eryptosis may be triggered by an increase in the cytosolic Ca^2+^ concentration, which may be due to Ca^2+^ influx via non-selective cation channels of the TRPC family. Eryptosis is further induced by ceramide, which sensitizes erythrocytes to the eryptotic effect of Ca^2+^. Signaling regulating eryptosis further involves a variety of kinases including AMPK, PAK2, cGKI, JAK3, CK1α, CDK4, MSK1/2 and casein kinase. Eryptosis-dependent shrinkage is induced by K^+^ efflux through Ca^2+^-activated K^+^ channel K_Ca_3.1, the Gardos channel. Eryptotic cells are phagocytosed and may adhere to endothelial cells. Eryptosis may help prevent hemolysis since defective erythrocytes usually undergo eryptosis followed by rapid clearance from circulating blood. Excessive eryptosis stimulated by various diseases and xenobiotics may result in anemia and/or impaired microcirculation. This review focuses on the significance and mechanisms of eryptosis as well as on the ion fluxes involved. Moreover, a short summary of further ion transport mechanisms of the erythrocyte membrane is provided.

## Introduction

The number of erythrocytes or red blood cells (RBCs) exceeds the numbers of most other cells in the human body and amounts to 4–6 × 10^6^ per μl of blood (Nemkov et al., 2018). With a total of approx. 5 l of blood, every human being has about 25 × 10^12^ circulating erythrocytes. The main function of erythrocytes is the transport of O_2_ from the lung to organs, tissues, and cells which they need for oxidative phosphorylation. Erythrocytes are perfectly adapted to this task in that they are full of hemoglobin, the O_2_-carrying molecule, without having any cell organelles such as mitochondria, ribosomes, or a nucleus. Therefore, the metabolism of erythrocytes is restricted to degradation of glucose without O_2_. They cannot utilize energy-rich fatty acids at all. Moreover, mature erythrocytes do not have RNA or DNA and cannot synthesize proteins. Owing to these limitations, they have simply been described as “bags of hemoglobin” rather than real cells (D’Alessandro and Zolla, 2017).

Normal cells can undergo apoptosis, a form of programmed cell death (D’Arcy, 2019). It allows cells to die in a controlled sequence of events that affect the cytosol and different organelles. For example, it comprises the breakdown of the mitochondrial potential, karyopyknosis (shrinkage of the nucleus and chromatin condensation) with subsequent DNA degradation, Ca^2+^ influx and Ca^2+^-dependent enzymatic digestion of intracellular proteins as well as breakdown of the phosphatidylserine asymmetry of the cell membrane (Guo et al., 2009; D’Arcy, 2019). The latter results in the appearance of phosphatidylserine in the outer membrane leaflet whereas it can only be found in the inner leaflet in non-apoptotic cells (Segawa and Nagata, 2015). Apparently, apoptosis as such is not possible in erythrocytes due to the lack of organelles. However, it has become clear that also erythrocytes, the lifespan of which has a median of 120 days in human beings, can actively undergo a controlled suicidal death program which is in many aspects comparable to apoptosis of nucleated cells (Föller et al., 2008b; Lang et al., 2008). Hence, it has been called eryptosis and is mainly characterized by two hallmarks that are also typical of apoptosis (Repsold and Joubert, 2018): The externalization of membrane phosphatidylserine at the cell surface and the loss of cell volume, i.e., cell shrinkage (Föller et al., 2008b; Lang et al., 2008). The Nomenclature Committee on Cell Death 2018 did not recommend use of the term “eryptosis” despite its “unquestionable relevance” for the reason that it is “extremely complex […] to define the death of entities that—in physiological conditions—exist in a debatable state between life and death (such as erythrocytes and viruses)” (Galluzzi et al., 2018). Nevertheless, we use the term “eryptosis” in this review because we believe that it well reflects the similarities to apoptosis of nucleated cells and at the same time points to the obvious limitations associated with erythrocytes being cells without organelles.

## Significance of Eryptosis

Erythrocytes may become damaged during their lifetime such that they are lysed and release hemoglobin (hemolysis) (Föller et al., 2008b). Although plasma protein haptoglobin binds free hemoglobin with high affinity, the lysis of a great number of erythrocytes can exceed its binding capacity (Shih et al., 2014). If so, plasma hemoglobin can freely be filtered in the kidney and may precipitate in kidney tubules resulting in kidney damage and ultimately in acute kidney injury (AKI), a condition with high mortality often requiring intensive care treatment (Qian et al., 2010). Eryptosis may be a mechanism to prevent this potentially life-threatening complication of hemolysis by initiating a suicidal death program in damaged red blood cells that provides for the controlled removal of the affected cell before the damage may cause uncontrolled hemolysis (Föller et al., 2008b). Along these lines, eryptosis is triggered by a broad spectrum of endogenous and exogenous noxious insults including (bacterial) toxins, pharmaceutical drugs, several clinical conditions and acute and chronic diseases, as well as further biotic and abiotic stressors including oxidative stress or hyperthermia (Lang and Lang, 2015).

Cells of the mononuclear phagocyte system (MPS) such as macrophages recognize phosphatidylserine on the surface of eryptotic cells by means of specific receptors (Bonomini et al., 2001; Mandal et al., 2002; Segawa and Nagata, 2015). This interaction prompts the phagocytosis of the dying erythrocytes, resulting in its engulfment and intracellular degradation. Hence, phosphatidylserine exposure in eryptosis serves as an “eat-me” signal (Bonomini et al., 2001; Mandal et al., 2002; Segawa and Nagata, 2015).

## Pathophysiology of Eryptosis

The presentation of phosphatidylserine on the surface of eryptotic cells can have two major pathophysiological implications: On the one hand, it initiates phagocytosis of erythrocytes (Mandal et al., 2002), on the other hand it mediates the adherence of erythrocytes to vascular endothelium cells which also express phosphatidylserine receptors (Setty and Betal, 2008; Wautier et al., 2011).

Excessive eryptosis initiating the phagocytosis of many red blood cells may therefore result in an acute loss of erythrocytes, i.e., anemia (Lang F. et al., 2017). In line with this, many of the aforementioned stimulators of eryptosis are associated with anemia, i.e., the pharmaceutical drugs triggering eryptosis are known to cause anemia as an adverse effect and the eryptosis-associated diseases are paralleled or even characterized by anemia (Lang and Lang, 2015).

The adherence of eryptotic erythrocytes to vascular endothelium cells also mediated by the phosphatidylserine receptor may impair microcirculation. Hence, stimulators of eryptosis may not only cause anemia, but also cardiovascular complications due to impeded microcirculation (Pretorius, 2018).

The induction of eryptosis may, however, have beneficial consequences, too: Malaria, a tropical disease threatening hundreds of million people world-wide and responsible for several hundred thousand deaths every year, is caused by protozoan *Plasmodium falciparum*, an unicellular eukaryote (Föller et al., 2009a; Boulet et al., 2018). During the infection, the pathogen invades erythrocytes and matures in the red cell, finally causing its lysis thereby releasing new parasites that can infect further erythrocytes. The lysis causes cyclical periods of fever, the hallmark of malaria (Föller et al., 2009a; Boulet et al., 2018). Given the dependence of the parasite on intraerythrocytic maturation, the early induction of eryptosis appears to be a promising therapeutic strategy as it could result in the phagocytosis of the affected erythrocyte and the inside parasite, thus in the clearance of the pathogen (Foller et al., 2008; Boulet et al., 2018). A big flaw of most common therapeutic approaches in malaria that target the parasite is the development of resistance. Therefore, the stimulation of eryptosis, a therapeutic strategy aiming at the host, could be helpful to prevent resistance (Foller et al., 2008; Boulet et al., 2018).

## Phosphatidylserine Externalization in Eryptosis

In erythrocytes, the activities of the enzymes scramblase and flippase (aminophospholipid translocase) determine the distribution of phosphatidylserine among the inner and outer membrane leaflet (Pretorius et al., 2016): Flippase translocates phosphatidylserine from the outer leaflet to the inner leaflet, thereby maintaining phosphatidylserine asymmetry in non-eryptotic cells (Pretorius et al., 2016). Conversely, scramblase shifts phosphatidylserine from inside to outside (Pretorius et al., 2016). Both enzymes are Ca^2+^-regulated with Ca^2+^ inhibiting flippase and activating scramblase (Weiss et al., 2012). Upon induction of eryptosis, an increase in the cytosolic Ca^2+^ concentration inhibits flippase and activates scramblase resulting in the appearance of phosphatidylserine on the surface of the dying erythrocyte.

In patients with sickle cell disease, phosphatidylserine externalization is found in subsets of reticulocytes, young cells, and partly also in mature erythrocytes (Jong et al., 2001). Interestingly, phosphatidylserine exposure can be reversed in sickle cell disease, particularly in young erythrocytes (Yasin et al., 2003).

## Cell Shrinkage in Eryptosis

Apart from externalization of phosphatidylserine, cell shrinkage is another hallmark of eryptosis (Föller et al., 2008b). The loss of cell volume is due to the efflux of K^+^ ions which is paralleled by efflux of Cl^–^ and osmotically obliged water (Föller et al., 2008b). Human erythrocytes express Ca^2+^-regulated inwardly rectifying K^+^ channels which are also known as Gardos channels (K_Ca_3.1) encoded by KCNN4 (Hoffman et al., 2003). The increase in the cytosolic Ca^2+^ concentration as an intitial event in eryptosis leads to the formation of Ca^2+^/calmodulin that is associated with the Gardos channel thereby activating it (Oliván-Viguera et al., 2013). At 2 μM Ca^2+^, Gardos channel activity is maximal (Thomas et al., 2011). Gardos channel-mediated K^+^ efflux hyperpolarizes the cell membrane. As a consequence, hyperpolarization drives anion exit through Cl^–^ channels (Dyrda et al., 2010). The cell volume loss upon Gardos channel activation is in large part rate-limited by efflux of Cl^–^ following K^+^ efflux (Thomas et al., 2011). In unstimulated erythrocytes, the Gardos channel as well as most other cation channels are closed resulting in a low cation leakage. A certain mutation of the KCNN4 gene enhances the Ca^2+^ sensitivity of the Gardos channel and accounts for hereditary xerocytosis, an autosomal-dominant disease (Rapetti-Mauss et al., 2015). It is characterized by dehydrated erythrocytes and hemolytic anemia (Rapetti-Mauss et al., 2015).

The activation of Gardos K^+^ channels with subsequent cell shrinkage is not only a hallmark of eryptosis but also enhances erythrocyte phosphatidylserine exposure stimulated by energy depletion or Ca^2+^ ionophore ionomycin (Lang P. A. et al., 2003). Hence, Gardos channel-mediated K^+^ loss is itself a trigger of eryptosis (Lang K. S. et al., 2003).

Patients with sickle cell disease have a subfraction of erythrocytes (app. 4%) that exhibit a relatively high intracellular Na^+^ and low intracellular K^+^ concentration (Bookchin et al., 2000). Such erythrocytes are also found in healthy humans (<0.03%) and do not dehydrate (“valinomycin-resistant”) when exposed to K^+^ ionophore valinomycin or a Ca^2+^ ionophore (Bookchin et al., 2000).

## Ca^2+^ Entry Into Erythrocytes

An increase in the cytosolic Ca^2+^ concentration is an early event in the orchestration of eryptosis and initiates hallmarks of eryptosis, phosphatidylserine externalization and cell shrinkage (Föller et al., 2009b). Under resting conditions in non-stimulated erythrocytes the cytosolic free Ca^2+^ concentration is in the range of 50 nM (Bogdanova et al., 2013), a value several orders lower than the free plasma Ca^2+^ concentration (around 1.5 mM) and comparable to the free cytosolic Ca^2+^ concentration in nucleated cells. Total cytosolic Ca^2+^ also includes Ca^2+^ ions bound by Ca^2+^-binding proteins such as calmodulin and amounts to 5–6 μM (Bookchin and Lew, 1980; Bogdanova et al., 2013).

Ca^2+^ entry into erythrocytes is incompletely understood. Clearly, the low basal Ca^2+^ conductance of unstimulated erythrocytes can be increased, suggesting the presence of Ca^2+^ channels in the membrane. These Ca^2+^-permeable channels exhibit a low conductance in non-stimulated erythrocytes (Duranton et al., 2002). They are regulated by the Cl^–^ concentration, as replacement of extracellular Cl^–^ by gluconate or other anions such as Br^–^, SCN^–^, or I^–^ strongly up-regulates their conductance (Duranton et al., 2002). Moreover, these Ca^2+^-permeable channels are activated by oxidative stress (Duranton et al., 2002) and hyperosmotic shock (extracellular osmolarity of 850 mOsm) and can be inhibited by ethylisopropylamiloride (Lang K. S. et al., 2003) and erythropoietin (Myssina et al., 2003). They have a permselectiviy of Cs^+^ > K^+^ > Na^+^ = Li^+^ >> NMDG^+^ (Duranton et al., 2002).

Activators of the cation channels include prostaglandin E_2_ (PGE_2_) (Lang et al., 2005). Phospholipase A2 cleaves phospholipids in the cell membrane, releasing arachidonic acid. Cyclooxygenase is the key enzyme for the generation of prostaglandins from arachidonic acid. Prostaglandin E_2_ (PGE_2_) is an important eicosanoid, contributing to pain by sensitizing peripheral nociceptors, to fever and local inflammation (Ricciotti and FitzGerald, 2011). The isosmotic replacement of extracellular Cl^–^ and hyperosmotic shock induce PGE_2_ synthesis in erythrocytes which in turn activates Ca^2+^ influx through the Ca^2+^-permeable cation channel (Lang et al., 2005). Hence, PGE_2_ is a major regulator of eryptosis by inducing Ca^2+^ influx (Lang et al., 2005).

Interestingly, ionotropic glutamate NMDA receptors permeable to Ca^2+^ contribute to Ca^2+^ homeostasis in erythrocytes (Makhro et al., 2013; Kaestner et al., 2020). Furthermore, human erythrocytes express GluA1, a subunit of ionotropic glutamate AMPA receptors that are also Ca^2+^ permeable if devoid of subunit GluA2 (Föller et al., 2009b). AMPA receptor antagonists attenuate the increase in cytosolic Ca^2+^ following removal of extracellular Cl^–^ or glucose, maneuvers inducing eryptosis by stimulating Ca^2+^ entry (Föller et al., 2009b).

TRP channels are a large family of cation channels permeable to different cations, expressed in many different cell types, and having a broad spectrum of physiological functions (Ramsey et al., 2006). TRPC2, TRPC3 and TRPC6 are Ca^2+^-permeable members of the TRPC family of channels expressed in erythrocyte precursor cells (Hirschler-Laszkiewicz et al., 2011, 2012). Erythropoietin induces Ca^2+^ entry into these cells through TRPC3 (Hirschler-Laszkiewicz et al., 2011). In mature human erythrocytes, TRPC6 may contribute to Ca^2+^ entry in eryptosis (Foller et al., 2008) whereas TRPC4/5 may be more relevant in mature murine erythrocytes (Danielczok et al., 2017).

Ca^2+^ entry into erythrocytes is also induced by shear stress (Larsen et al., 1981; Johnson and Gannon, 1990; Thomas et al., 2011). Erythrocytes express the mechanosensitive cation channel PIEZO1 (see below) permeable to Ca^2+^ (Cahalan et al., 2015). Indeed, mechanical stress induces PIEZO1-dependent Ca^2+^ influx into erythrocytes that, in turn, stimulates Gardos channel-mediated K^+^ efflux and shrinkage (Cahalan et al., 2015).

Human erythrocytes also express voltage-dependent Ca_*v*_2.1 Ca^2+^ channels as confirmed by Western Blotting (Andrews et al., 2002). Pharmacological inhibition of Ca_*v*_2.1 with ω-agatoxin-TK suppresses Ca^2+^ influx into erythrocytes induced by phorbol 12-myristate 13-acetate (PMA) (Andrews et al., 2002). According to this study, PKC regulates Ca_*v*_2.1-mediated Ca^2+^ entry into erythrocytes (Andrews et al., 2002). This view is challenged by another study postulating that at least two different Ca^2+^ influx pathways exist in erythrocytes with one being independent of Ca_*v*_2.1 and another indirectly activated by PKCα (Wagner-Britz et al., 2013). PKCα also regulates eryptosis induced by energy depletion (Klarl et al., 2006).

## Ca^2+^ Atpase

The plasma membrane P-type Ca^2+^ ATPase (PMCA) is expressed in human erythrocytes (Bogdanova et al., 2013). It is equipped with a Ca^2+^/calmodulin binding site interacting with Ca^2+^/calmodulin and enabling it to sense an elevation of the cytosolic Ca^2+^ concentration (Bogdanova et al., 2013). In this case, it pumps Ca^2+^ out of the cell at the expense of ATP which is degraded by the enzyme (Bogdanova et al., 2013). Ceramide or arachidonic acid induce the activity of the Ca^2+^ ATPase (Bogdanova et al., 2013).

## Role of Ceramide and Kinases in Eryptosis

Apart from intracellular Ca^2+^, ceramide is a major cellular initiator of eryptosis (Lang et al., 2004). Ceramide is generated by sphingomyelinase, a phosphodiesterase that cleaves membrane sphingolipid sphingomyelin (Zeidan and Hannun, 2010). Ceramide is involved in apoptosis of nucleated cells mediated by the death receptor CD95 (Gulbins et al., 1995). It triggers eryptosis by sensitizing the cell to Ca^2+^, but it does not induce Ca^2+^ influx (Lang et al., 2004). Moreover, hyperosmotic shock induces eryptosis also by stimulating sphingomyelinase-dependent ceramide formation in erythrocytes, an effect explaining why hyperosmotic shock-stimulated eryptosis is also observed in the absence of extracellular Ca^2+^ (Lang et al., 2004).

Regulators of eryptosis further include diverse kinases including AMP-activated protein kinase AMPK (Föller et al., 2009c; Zelenak et al., 2011), p21-activated kinase PAK2 (Zelenak et al., 2011), cGMP-dependent protein kinase type I cGKI (Föller et al., 2008a), Janus kinase JAK3 (Bhavsar et al., 2011), casein kinase CK1α (Kucherenko et al., 2012; Zelenak et al., 2012), cyclin-dependent kinase CDK4 (Lang et al., 2015b), and mitogen- and stress-activated kinase MSK1/2 (Lang et al., 2015a) as suggested by pharmacological approaches and/or experiments with knockout mice.

## Role of Oxidative Stress in Eryptosis

Erythrocytes are permanently challenged by oxidative stress (Cimen, 2008). Despite having powerful reactive oxygen species (ROS) scavengers including glutathione, superoxide dismutase, or catalase, ROS is an important trigger of eryptosis *in vivo*, especially in certain clinical conditions (Lang et al., 2014). In mice deficient for the modifier subunit of glutamate–cysteine ligase (gclm^–/–^ mice), the erythrocyte glutathione level is only 10% of the normal value (Föller et al., 2013). Nevertheless, no enhanced eryptosis is observed in unchallenged mice. Upregulated catalase may contribute to this effect (Föller et al., 2013). If the mice are, however, exposed to additional oxidative stress in the form of phenylhydrazine, eventually fatal hemolysis is the consequence (Föller et al., 2013).

Ca^2+^-permeable cation channels initiating eryptosis are activated by ROS (Duranton et al., 2002), and higher *in vitro* susceptibility of erythrocytes from gclm^–/–^ mice to eryptosis can be blocked by antioxidant Trolox (Föller et al., 2013). In addition, erythrocyte Cl^–^ channels contributing to shrinkage in eryptosis are also sensitive to ROS (Huber et al., 2002).

Oxidative stress may contribute to enhanced eryptosis in several clinical conditions including diabetes, chronic kidney disease, Wilson’s disease, malaria, and iron deficiency (Lang et al., 2014). In erythrocytes from patients with sickle cell anemia, antioxidants inhibit K^+^, Cl^–^ cotransport, and Gardos channel-mediated K^+^ efflux as well as phosphatidylserine exposure (Al Balushi et al., 2019). Hence, also the erythrocyte K^+^ permeability is dependent on ROS, at least in erythrocytes from patients with sickle cell disease (Al Balushi et al., 2019).

A recent study uncovered that lysates of erythrocytes contain a vast number of pro-inflammatory and anti-inflammatory cytokines, chemokines, and mediators including C-C chemokines (CTACK, Eotaxin, MCP-1, MCP-3, MIP-1α, MIP-1β, RANTES), members of the CSF family (G-CSF, GM-CSF, M-CSF), C-X-C chemokines (GRO-α, IL-8, IP-10, MIG, SDF-1α), FGF growth factors (bFGF), IL-3, IL-5, IFNα2, IFNγ, members of the IL-1 family, LIF, IL-12(p40), IL-12(p70), IL-17, MIF, PDGF-bb, VEGF, TNFα, TNFβ, and TRAIL (Karsten et al., 2018). Whether or not these mediators are involved in the regulation of eryptosis should be addressed in future investigations.

**FIGURE 1:**
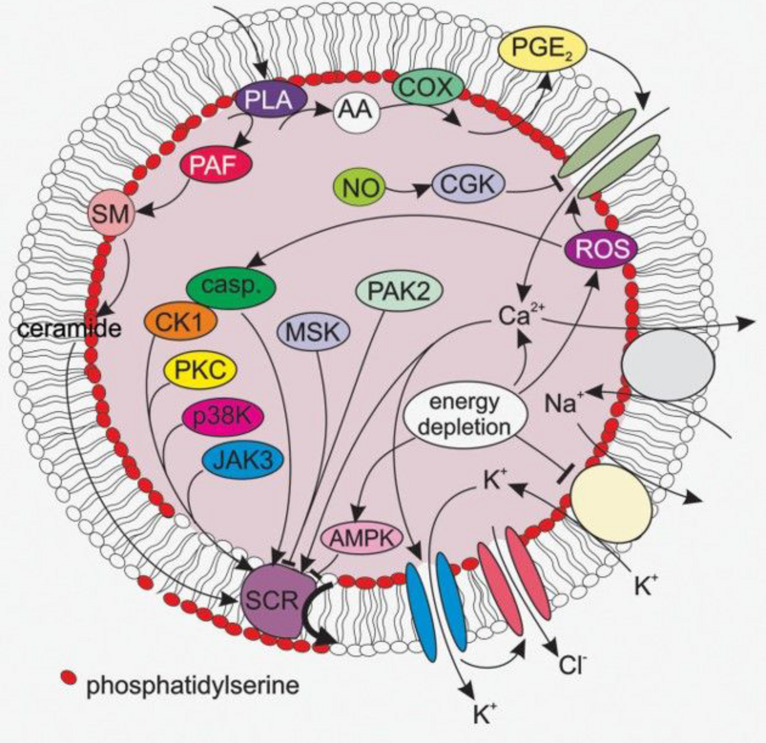
A selection of signaling pathways relevant for eryptosis. AA, arachidonic acid; AMPK, AMP-activated kinase; casp, caspases; cGK, cGMP-dependent protein kinase 1; CK1, casein kinase 1; COX, cycloxygenase; JAK3, janus kinase 3; MSK, mitogen- and stress- activated kinase; PAK2, p21-activated kinase 2; p38 MAPK, p38 mitogen-activated protein kinase; PAF, platelet activating factor; PGE2, prostaglandin E2; PKC, protein kinase C; PLA, phospholipase A; SCR, scramblase; SM, sphingomyelinase. The figure was taken from the review by Lang E. et al. (2017).

A selection of important mechanisms of eryptosis is summarized in Figure 1.

## Further Ca^2+^-Dependent Processes in Erythrocytes

An elevation of the cytosolic Ca^2+^ concentration results in lower O_2_ affinity of hemoglobin (Bogdanova et al., 2013; Makhro et al., 2013).

Endothelial NO synthase (eNOS)-dependent NO production is stimulated by an increase in the cytosolic Ca^2+^ concentration (Ulker et al., 2011; Bogdanova et al., 2013). By the same token, NO inhibits eryptosis (Nicolay et al., 2008).

Calpain 1 (μ-calpain) is a cysteine protease that is activated by an elevation of the intracellular Ca^2+^ concentration (Bogdanova et al., 2013). It is expressed in human erythrocytes (Hatanaka et al., 1984) and degrades membrane-associated proteins (Bogdanova et al., 2013). Calpain is likely to contribute to the breakdown of proteins in eryptosis (Lang et al., 2006).

## Further Ion Transport Mechanisms in Erythrocytes

Erythrocytes express the mechanosensitive non-selective cation channel PIEZO1 which is stretch-activated (Zarychanski et al., 2012; Bae et al., 2013) and opens upon mechanical stress also allowing cell volume loss (Badens and Guizouarn, 2016). Mutations of the FAM38A gene encoding PIEZO1 are responsible for hereditary xerocytosis (Zarychanski et al., 2012; Bae et al., 2013).

The anion exchanger 1 (AE1 or SLC4A1) is encoded by the SLC4A1 gene and is also known under the name Band 3 (Abbas et al., 2018). The two different names hint at distinct functions: It is the most abundant protein of the erythrocyte membrane and part of its cytoskeleton by interacting with ankyrin or band 4.2, other cytoskeleton proteins (Kümpornsin et al., 2011). As an anion exchanger, it mediates the electroneutral exchange of Cl^–^ ions for HCO_3_^–^ ions which is part of the mechanism of CO_2_ transport from peripheral tissues and organs to the lung (Abbas et al., 2018). In this respect, it is also known as Hamburger phenomenon or chloride shift (Colombo et al., 2020). Several mutations of the SLC4A1 gene are described which result in Na^+^ and/or K^+^ leakage (Guizouarn et al., 2007; Ellory et al., 2009; Barneaud-Rocca et al., 2013; Reithmeier et al., 2016). This phenomenon is not fully explained and may be due to the mutation changing AE1 into a cation channel or activating other cation channels in the erythrocyte membrane (Guizouarn et al., 2007; Ellory et al., 2009; Barneaud-Rocca et al., 2013).

In erythrocytes infected with *P. falciparum* endogenous inwardly and outwardly rectifying Cl^–^ channels are activated (Huber et al., 2002). Moreover, similar anion conductances can be induced by oxidative stress (Huber et al., 2002). Hyperpolarization-activated CLCN2 chloride channels in the erythrocyte membrane contribute to this anion conductance of *Plasmodium berghei*-infected mouse erythrocytes and participate in cell volume regulation (Huber et al., 2004). These channels exhibit a 5 pS conductance and are inhibited by Zn^2+^ (Thomas et al., 2011). They are small conductance chloride channels (SCC) characterized by alternating long-lasting phases of opening and closing (Thomas et al., 2011).

Maxi-anion channels with a conductance of several hundred pS up to the nS range have been suggested to be involved in anion transport across the erythrocyte membrane (Glogowska et al., 2010). According to this study, they are not active *per se*, but can be induced by serum components and exhibit multifaceted gating mechanisms and kinetics (Glogowska et al., 2010).

An erythrocyte anion channel with particular relevance in malaria infection is part of the family of voltage-dependent anion channels (VDAC), presumably VDAC3 (Thomas et al., 2011). In the erythrocyte membrane, VDAC is part of a peripheral-type benzodiazepine receptor complex which is in addition made up by adenine nucleotide transporter (ANT) and a translocator protein (TSPO) (Thomas et al., 2011). This peripheral-type benzodiazepine receptor complex may be the molecular correlate for the maxi-anion channel suggested by a previous study (Glogowska et al., 2010). However, whether or not maxi anion channels are identical to VDACs is controversially discussed (Pinto et al., 2010). In *Plasmodium*-infected erythrocytes, the peripheral-type benzodiazepine receptor complexes involving VDAC are up-regulated and may contribute to the “new permeability pathways” that are induced in erythrocytes by the malaria pathogen and required for their intraerythrocytic survival (Bouyer et al., 2011). In line with this, pharmacological inhibition of the peripheral-type benzodiazepine receptor compromises the growth of the pathogen (Bouyer et al., 2011).

Also the gene encoding glucose carrier GLUT1 which accomplishes glucose uptake by erythrocytes can be affected by a mutation that renders the carrier into a cation channel with Na^+^, K^+^ as well as Ca^2+^ conductance. Simultaneously, glucose transport is impaired (Weber et al., 2008).

The Rhesus-associated glycoprotein (RHAG) is an ammonia (NH_3_) carrier (Ripoche et al., 2004). Importantly, it is part of the Rhesus blood group system and can transport NH_3_, NH_4_^+^, as well as CO_2_. Mutations of the gene encoding RHAG account for overhydrated stomatocytosis characterized by hemolytic anemia and red blood cells with an enhanced cation leakage which is higher for Na^+^ than for K^+^ (Stewart et al., 2011). As a consequence, the erythrocytes are swollen (overhydrated) due to an elevated intracellular Na^+^ concentration that, in turn, enhances Na^+^/K^+^ ATPase activity. The latter results in higher need for ATP which is generated in glycolysis (Darghouth et al., 2011).

From electrophysiolgical recordings (patch clamping), the expression of a low conductance cation channel in erythrocytes was concluded in early studies (Kaestner, 2011). It is supposed to have a conductance of 8–17 pS (Kaestner, 2011).

It is yet unknown whether and to which extent the further ion transport mechanisms presented in this section are required for the cellular machinery initiating and executing eryptosis. Clearly, future studies are needed to address this issue.

## Perspectives

Ion transport in eryptosis is still incompletely understood. Future research should address the molecular identity of the channels and transporters involved. The specific pharmacological targeting of ion transport mechanisms required for the execution of eryptosis may turn out to be favorable in different clinical conditions including anemia, impaired microcirculation, or malaria.

## Author Contributions

MF and FL wrote the review. Both authors contributed to the article and approved the submitted version.

## Conflict of Interest

The authors declare that the research was conducted in the absence of any commercial or financial relationships that could be construed as a potential conflict of interest.
